# Preservation of Morphia Solutions

**Published:** 1875-12

**Authors:** 


Preservation of Morphia Solutions.—It is asserted by M. Vidal, that the addition of chloral to a solution of morphia renders it much less liable to spontaneous change. That fact, if it be true, is important. The alteration which concentrated solutions of morphia undergo renders their strength variable and uncertain if they are laid by for a time. M. Vidal adds to the solution a quantity of chloral equivalent to twice the weight of the morphia it contains. He affirms that the injection of this mixture is not painful.—*The Medical Record.*

